# Implementation of a psychoeducational program to reduce depressive and anxiety symptoms in new mothers: preliminary results of a clinical trial

**DOI:** 10.1192/j.eurpsy.2026.11839

**Published:** 2026-07-31

**Authors:** A. Roca Lecumberri, N. Fernandez, S. Albero, L. Lucas, M. J. Cid, B. Albillos, J. Pelegrí, E. Vela, E. Gelabert

**Affiliations:** 1 Perinatal Mental Health Unit, Hospital Clínic Barcelona; 2 Atenció Salut Sexual i Reproductiva (ASSIR); 3 Department of Clinical and Health Psychology, Universitat Autonoma de Barcelona, Barcelona, Spain

## Abstract

**Introduction:**

Perinatal mental disorders represent a public health issue due to their impact on both women’s and children’s health. It is estimated that one in five women will experience a mental disorder during perinatal period. Several randomized controlled trials have tested interventions to prevent PMD, ranging from selective interventions for at-risk women to universal interventions for all women with contradictory results. Our study proposes a brief, two-session psychoeducational intervention delivered by midwives during the postnatal period.

**Objectives:**

To reduce anxiety and depressive symptoms in new mothers and improve the relationship with babies, and the perception of self-efficacy towards the care of the baby.

**Methods:**

Design: A randomized controlled trial of parallel groups was conducted in accordance with CONSORT requirements.

Participants: All primiparous women who had given birth to a healthy, full-term baby, over 18 years of age, and gave their consent to participate in the study were eligible to participate.

Procedures: Sociodemographic, obstetrical and clinical variables were collected at baseline. The following questionnaires were administered at baseline and 3 months follow-up: Edinburgh Postnatal Depression Scale (EPDS), Generalised Anxiety Disorder (GAD-7), Postpartum Bonding Questionnaire (PBQ) and Me as a parent (MaaP).

- Intervention Group: A psychoeducational program was delivered by midwives in primary care centers. It consists of two group sessions (total of 6 hours) within the first 6 weeks postpartum.
- Usual care Group: Participants receive the postpartum usual care at ASSIR (postnatal care visits, postpartum support groups).

Comparisons were made using nonparametric analyses, and related samples were compared using the Wilcoxon test.

**Results:**

The results of the first 100 women recruited for the study are presented (50 for each group).

Sociodemographic, obstetric, and clinical variables did not differ significantly between the two groups. No significant differences were found between the two groups on the self-administered scales at baseline or at the 3-month follow-up. The intervention group showed significant differences between baseline scores and scores at the 3-month on the EPDS (p .001) and the MaaP (p .002). The control group showed no significant differences.

Satisfaction surveys showed that both participants and midwives were satisfied with the intervention program, with an average score of 9/10.

**Conclusions:**

Preliminary results show a possible positive effect of psychoeducational intervention in reducing depressive symptoms and improving perceptions of maternal self-efficacy. Brief psychoeducational interventions tailored to the participating populations (for example primiparous mothers) may be useful for the prevention and improvement of maternal mental health and will be well accepted.

**Disclosure of Interest:**

A. Roca Lecumberri Grant / Research support from: Fundació la Marató: 193/C/2022, N. Fernandez: None Declared, S. Albero : None Declared, L. Lucas: None Declared, M. J. Cid: None Declared, B. Albillos: None Declared, J. Pelegrí: None Declared, E. Vela: None Declared, E. Gelabert Grant / Research support from: Fundació la Marató: 193/C/2022

